# Supplementary material for: Structural and Functional Dissection of the 5′ Region of the Notch Gene in Drosophila melanogaster
Source: Genes (Basel). 2019 Dec 12;10(12):1037. doi: 10.3390/genes10121037 (PMC6947440; doi:10.3390/genes10121037)
Supplement: Supplementary file 1 [file genes-10-01037-s001.pdf]

Supplementary

A

Genomic DNA / attP / loxP / Spacer

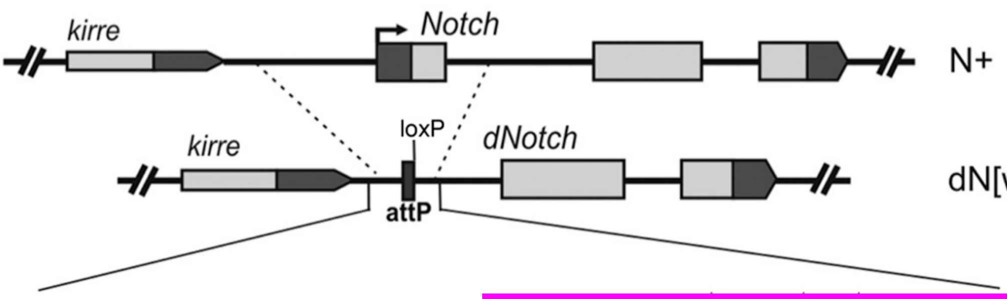

...ATGGCTGAGCTCGATGAGTTTCGCAGCGGTACCGTAGTGCCCCAACTGGGGTAACCTTTG
AGTTCTCTCAGTTGGGGGCGTAGTGTACCATAACTTCGTATAATGTATGCTATACGAAGTTA
TCACTAGTAAAGATCTCCATGCATAAGGCGCGCCATATGGAGCGGTCGTTGTCTAATCACT
G...

B

d4 /N$^{fa\text{-}swb}$/dfa-swb/ [d1]/d2/d3/ N exon1TAD-SextonTAD-Hou

ClaI

...GCAATTTAATCGATAATCCCCAAGCCGCAAAAAACTAAAAACAAAAAGATGTCCAAATATTGGTGATTTACGAGT
AAAGTGAATAAAATTAGAAAAACCACCAACATATATATATATATATATATATATAGTTCCCACTTTCCAACTTTTTG
ATCGGGTTTTCAGTGGCACAGCATTTCAGCATCCATGTGAAAAGTTGAATATTTTTTTGTCTAGCCCTAAGCGCGTAA
ATGATATTTGAACTTAAATCAATTATGTAAAGAAGCAAATGAAATATTGTATATCGTATATATATATATATATATAT
ATATATAGAGCATAAATGTGTATGTCAACGCTTGAATTAAAAAATGACTAGCTAAGATTAGAGAGAATGTTTGTAAT
TTTTTTTTTTGTCTGCCTAGTTTAGGTTTAAGCCTATTTATAGACGTAAATGAAGAATTTTAAAGGTAAAAAGAAAA
ACTTAAAAAGCTTAGAAACAATTTGTAAAACATT

TGAGCGATCTACCAAAAAAAAAAAAAACGCAATTAAGTGTAAAGCGTTCAATTTTTAACACGAAATGCTGCCTTAT
GATTCCT

PacI

CGTTGGGTTCTTAATTCACTAAGCCAATACTGTAATTAATTAACTAATTTATTGTTTCATATTTTATTTTTTGTTTCTTT
CGAGAATATACATATGTATGTATTTATCTAAAGTAATTCTGAAAATGCTTAAAAATAATGCCCGTTCGTTTAAACTG
ATTTATACACTCGAATCTAATTCTATTCTTTCCGTGGGCCAATTGCGCCGAGCTTTTTAACTGGGATAGCGTTCCGAA
AATCGGAGATAAGTATGGTGTTACATATTTTTTTTTGTTTTCGATAATTGTCAAACGATTCAATTAAGCGTTCAATTA
TATAGACGTTACACACGAAACGAAATGAAATTCAAATGAAATCCTATTAAGCCAAGTAAAGTATGATAATTGCAGG
AAGCGGAGTTGCGAAAA[AAAAAGGTACGGAAAATGAAAACTAAGAACGTATTGCGGGAAAACCTAACTTAAGTC
GAGAACAACTCAAATTAGTTGAAATAAAGATACGAGAACAAAAGGAAAATAAAATACTTAAAAAATGCTACAAGT
GCGTTTTTCAATCAAATTTATGCACATTTTTTTCCAGTGCCTACGAATTTTACATTGTCGAATTTTACATTATACATAG
CGTAATTTCTACGAATCTTACATTGTCGAATTTTACATTTTACATAGCGTAATTTCTACAT]ACCGCTATGACGGCAC
TAAAGCGCCATTCGGCGAAATGGGGAAACTACTCATGCAAGCGGCTCGGAGC

BssHII

CCGGCTAACGTTATTTGTTCACCAACCGATCTCGCAACGCTGCGAAAGAGCGCGCTGCCAAAATGGCTCCCCGCCAT
ACGGTATCTTTTTCTTGCACCGACGCGGTCACACTGCCGATTTGAAACAGATCGCTTTTTTCCAGTGGACGAAACGG
TTGTGAAAGCGGACGAGCGTTAGGCAGACGAACCTGGAAAGCGCAGAGCACAGTTCTCAACATTTATTTTTTTTTG

AATGTGTGTGCAACAACGCACGTAAAATCGCGCTGCCAACAGGATATACAAACAAATCAATTACACAGCAAGCAAA
TGCAATGAAATGAAAAGGATGGCCCCAGCGGGAAAGCCGTTCAGCAAGAGCAAGGAGTGCCTGTCGCAGGGATAG
CAACGAGAGAGCGACACAGAGAGCGAGA **XhoI**

GAGAGAGAGGGAGAGAAACAAGGATTTTCGAAAAGTGTATCTACCTCGAG…

C

**wt**	…GAGCGCGCTGCC|AAAATGGCTCCC…	Viable
**N$^{fa\text{-}swb}$**	…GAGCGATCTA-AAACGCA|AAAATGGCTCCC…	Viable
**dfa-swb^{LK}**	…GAGCGATCTA-AAACGCA|AAAATGGCTCCC…	Viable
**dfa-swb**	…GAGCGATCTAC|AATGGCTCCC…	Lethal
**d3**	…GTGCGTTTTTCAATCA|AATGGCTCCC…	Viable

Figure S1. Nucleotide sequences of targeted mutations in the 5′ end of *Notch* locus. (**A**) Scheme of the locus including the founder deletion dN replaced with the attP and loxP sites. The deleted region is pointed by vertical dashes. The sequences of the functional elements are shown in colored and shaded letters according to the legend (top). (**B**) Positions of the targeted mutations, exon 1, and formalized TAD boundary sites (Hou et al., 2012; Sexton et al., 2012) in this region are shown in colored and shaded letters according to the legend (top). Restriction sites are underlined. Putative CAT-box and transcription start sites are indicated by the double underline and wavy lines, respectively. (**C**) The genomic DNA junction sites of the deletions and viability of flies homozygous for these deletions in the absence of AEs are shown. The junction points of the deleted DNA sequences are shown with vertical lines; a putative CAT-box is underlined, its modified variants in deletions are marked with dashed underlines.

Table S1. Phenotypes alleles of *Notch* gene obtained

**Dominant alleles**	**Phenotype of hemizygous males**	
dN[w+]	lethal,<br>geterozygous females has nicked wings	
dN[w-]	lethal,<br>geterozygous females is normal	
**Recessive alleles**		**Phenotype of transgeterozygous females vs dN[w-]**
N-resc[w-]	+	+
d1[w-]	+	+
d2[w-]	+	+
d3[w-]	+	+
d4[w-]	+	+
N-resc[w+]	R-, N	R, VTN, Ma
d1[w+]	R-	R, VTN, Ma
d2[w+]	+	R, VTN, Ma
d3[w+]	RG	RG, VTN, H, Ma, C
d4[w+]	+	R, VTN, Ma
dfa-swb[w+]	lehtal	ND
dfa-swb[w-]	lethal	ND
dfa-swbLK[w+]	lehtal	ND
dfa-swbLK[w-]	RG	RG, VN, H, Ma, C

Notes: R= rough eyes; RG= rough and glossy eyes; H= phenotype "hairy" - extra and misaligned bristles on thorax and legs; Ma= additional bristles on the scutellum; V= thickened wing veins forming deltas at margin; T= gaps of triplo-row, N= nicked wings, C = curved tibia of hind legs; **+** indicates normal phenotype, **-** indicates the expression is slight or is variable, overlapping wild type.
